# Safety and Immunogenicity of Concomitant Administration and Combined Administration of Bivalent BNT162b2 COVID-19 Vaccine and Bivalent RSVpreF Respiratory Syncytial Virus Vaccine with or Without Quadrivalent Influenza Vaccine in Adults ≥ 65 Years of Age

**DOI:** 10.3390/vaccines13020158

**Published:** 2025-02-05

**Authors:** Joel M. Neutel, Rahsan Erdem, Qin Jiang, Kevin Cannon, Helen Stacey, Ryan Newton, Emily Gomme, Wen Li, Federico J. Mensa, Özlem Türeci, Uğur Şahin, Kena A. Swanson, Iona Munjal, David Cooper, Kenneth Koury, Annaliesa S. Anderson, Alejandra Gurtman, Nicholas Kitchin

**Affiliations:** 1 Orange County Research Center, Santa Ana, CA 92630, USA; 2 Vaccine Research and Development, Pfizer Inc., Pearl River, NY 10965, USA; emily.gomme@pfizer.com (E.G.); iona.munjal@pfizer.com (I.M.);; 3 Vaccine Research and Development, Pfizer Inc., Collegeville, PA 19426, USA; 4 Accellacare, Wilmington, NC 28401, USA; 5 Diablo Clinical Research, Walnut Creek, CA 94598, USA; 6 Vaccine Research and Development, Pfizer Ltd., Marlow SL7 1YL, UK; ryan.newton@pfizer.com (R.N.); nicholas.kitchin@pfizer.com (N.K.); 7 BioNTech, 55131 Mainz, Germany

**Keywords:** BNT162b2, concomitant, immunogenicity, RSVpreF, QIV, noninferiority, safety

## Abstract

Concomitant administration may improve vaccination rates. This analysis of a phase 1/2 randomized study included 1073 healthy ≥65-year-olds who previously received ≥3 mRNA COVID-19 vaccine doses. Participants received concomitantly administered RSVpreF and bivalent BA.4/BA.5-adapted BNT162b2 vaccine (concomitant administration) with or without quadrivalent influenza vaccine (QIV), admixed combined RSVpreF + BNT162b2 vaccine (combined vaccine) with or without QIV, RSVpreF, BNT162b2, or QIV. Immunogenicity objectives included demonstrating the noninferiority of neutralizing antibody titers elicited by concomitant administration and combined vaccine compared with RSVpreF or BNT162b2 administered alone, and by concomitant administration and combined vaccine given with QIV compared with RSVpreF, BNT162b2, and QIV alone. Reactogenicity (≤7 days) and safety ≤1 month (adverse events (AEs)) and ≤6 months (serious AEs (SAEs)) after vaccination were assessed. Noninferiority for all immunogenicity comparisons was demonstrated. All vaccine groups were well tolerated; no new safety concerns were identified. Reactogenicity was mostly mild/moderate with rates generally similar across groups, except injection site pain and fatigue, which were less frequent with RSVpreF + placebo vs. other groups. AEs were infrequent, mostly mild/moderate, occurring at similar frequencies across groups. No AEs leading to study withdrawal or vaccine-related SAEs were reported. Favorable safety and tolerability alongside similar immunogenicity provide support for concomitant or combined use of RSVpreF and BNT162b2, with or without QIV, to help protect older adults from these important respiratory pathogens (NCT05886777).

## 1. Introduction

Seasonal patterns, typically in winter months, are well documented for several respiratory viral diseases [1,2], including those caused by respiratory syncytial virus (RSV) and influenza, which burden healthcare systems and cause appreciable mortality and morbidity [3,4]. RSV-associated illness results in considerable burden in older adults, with an estimated 100,000 to 150,000 hospitalizations occurring annually in the United States among those 60 years and older [5]. Similarly, influenza causes substantial burden in older adults, with the 2017 Global Burden of Disease Study estimating mortality rates from influenza-associated lower respiratory tract illness (LRTI) were highest among adults older than 70 years (16.4 deaths per 100,000) [4]. Although SARS-CoV-2 transmission occurs throughout the year, COVID-19 cases and associated hospitalization and mortality appear to peak during the winter season [6]. As with RSV and influenza, COVID-19 disproportionately affects older adults [7]; in the United States from January to August 2023, individuals 65 years and older accounted for more than 60% of all COVID-19-associated hospitalizations and nearly 90% of deaths during these hospitalizations [8].

The RSV bivalent prefusion F protein-based vaccine (RSVpreF; ABRYSVO™, Pfizer Inc, New York, NY, USA) contains stabilized prefusion F glycoproteins from RSV-A and RSV-B, the two cocirculating antigenic subgroups [9,10]. RSVpreF is licensed for the prevention of RSV-associated LRTI in adults 60 years and older [10,11]. The original BNT162b2 COVID-19 mRNA vaccine (COMIRNATY^®^; BioNTech Manufacturing GmbH and Pfizer Inc., New York, NY, USA) was approved in 2021 as a two-dose primary series [12]. With emerging SARS-CoV-2 variants differing substantially from the ancestral strain targeted by the original vaccine [13], variant-adapted versions of the BNT162b2 vaccine have since been approved. The variant-adapted version of BNT162b2 used in this study encoded original SARS-CoV-2 and Omicron BA.4/BA.5 spike proteins [14].

With SARS-CoV-2, RSV, and influenza infections coalescing in the autumn and winter seasons, keeping vulnerable populations up-to-date with vaccinations is critical to achieve maximal protection [1]. Concomitantly administering or combining RSV and COVID-19 vaccines could reduce the number of healthcare visits for older populations and thereby potentially improve vaccination rates. Given seasonal overlap of SARS-CoV-2, RSV, and influenza, administration of a seasonal influenza vaccine at the same time would further reduce healthcare visits. A study investigating the safety and immunogenicity of RSVpreF when concomitantly administered with seasonal inactivated influenza vaccine (SIIV) compared with administration of either vaccine alone supported coadministration of RSVpreF and SIIV in adults 65 years and older [15]. Similarly, another clinical study of the safety and immunogenicity of BNT162b2 concomitantly administered with SIIV supported coadministration of BNT162b2 with SIIV in adults 18 to 64 years of age [16].

The aim of this study was to evaluate the safety, tolerability, and immunogenicity of concomitantly administered and combined RSV and COVID-19 vaccines given with and without seasonal quadrivalent influenza vaccine (QIV) in older adults.

## 2. Materials and Methods

### 2.1. Design and Participants

This was a phase 1/2, randomized study (ClinicalTrials.gov identifier: NCT05886777) describing the safety, tolerability, and immunogenicity of concomitantly administered RSVpreF and BNT162b2 and a combined RSVpreF plus bivalent BNT162b2 (original/Omi BA.4/BA.5) vaccine (combined (RSVpreF + BNT162b2) vaccine) administered together with seasonal QIV (Fluzone^®^ HD; Sanofi Pasteur Inc., Swiftwater, PA, USA) or placebo (0.9% NaCl solution for injection). The admixed combined (RSVpreF + BNT162b2) vaccine comprises 120 µg RSVpreF containing two tabilized RSV prefusion F antigens (60 µg each of RSV-A and RSV-B) and 30 µg bivalent BNT162b2 (15 μg original/Omi BA.4/BA.5), which contains original BNT162b2 and BNT162b2 Omicron (15 μg B.1.1.529 sublineage BA.4/BA.5). The admixed combination was generated by mixing the individual vaccines at the study site in the specific dose-level combination.

The study included healthy adults 65 years and older, who had received at least 3 previous mRNA COVID-19 vaccine doses, with the last dose being a bivalent vaccine given at least 150 days before administration of the study vaccine. Participants should not have received any previous RSV vaccine at any time before enrollment or an influenza vaccine up to 120 days before enrollment. Further eligibility criteria are provided in the Supplementary Materials.

The study was conducted in accordance with the consensus ethical principles derived from international guidelines, including the Declaration of Helsinki and CIOMS International Ethical Guidelines, applicable ICH GCP guidelines, and applicable laws and regulations including applicable privacy laws. All participants provided written informed consent before enrollment.

### 2.2. Randomization, Interventions, and Blinding

**Figure 1:**
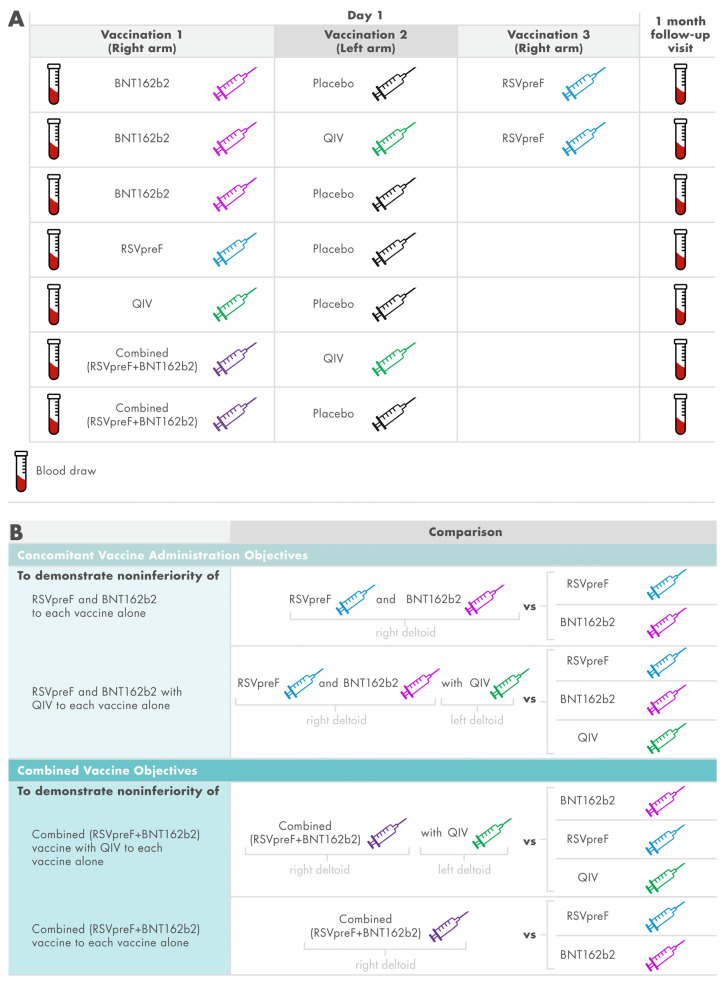
Study design (**A**) and objective and endpoints (**B**). HAI, hemagglutination inhibition assay; NT, neutralizing titer; QIV, quadrivalent influenza vaccine. In the first 2 groups, administration sites on the right arm were separated by 2.5 cm. Endpoints for the comparisons were RSV-A, RSV-B NTs (for RSVpreF); SARS-CoV-2 Omicron BA.4/BA.5 and ancestral-strain NTs (for BNT162b2); HAI titers for each strain contained in the QIV (for QIV).

Participants were randomized using interactive response technology to 1 of 7 study groups (Figure 1A). Participants in the 2 concomitant vaccine administration groups received either RSVpreF and BNT162b2 in the right arm separated by 2.5 cm and placebo in the left arm (i.e., RSVpreF/BNT162b2 group) or RSVpreF and BNT162b2 in the right arm separated by 2.5 cm and QIV in the left arm (i.e., RSVpreF/BNT162b2 with QIV group). Participants in the 3 single vaccine administration groups received either RSVpreF in the right arm and placebo in the left arm (i.e., RSVpreF group), BNT162b2 in the right arm and placebo in the left arm (i.e., BNT162b2 group), or QIV in the right arm and placebo in the left arm (i.e., QIV group). Participants in the 2 combined vaccine groups received either combined (RSVpreF + BNT162b2) vaccine administered in the right arm and QIV in the left arm (i.e., combined (RSVpreF + BNT162b2) with QIV group) or combined (RSVpreF + BNT162b2) in the right arm and placebo in the left arm (i.e., combined (RSVpreF + BNT162b2) group).

Participants were blinded to their assigned study intervention. Study staff receiving, storing, dispensing, preparing, and administering study interventions were unblinded. All other study and site personnel, including investigators and individuals evaluating participant safety, were blinded.

### 2.3. Immunogenicity Objectives and Assessments

Blood samples were collected before and 1 month after vaccination for immunogenicity assessments. Immunogenicity objectives and endpoints are summarized in Figure 1B. A primary immunogenicity objective was to demonstrate that immune responses elicited by the combined (RSVpreF + BNT162b2) vaccine were noninferior to those elicited by RSVpreF and BNT162b2 administered alone. The other primary immunogenicity objective was to demonstrate that immune responses elicited by the combined (RSVpreF + BNT162b2) vaccine given with QIV were noninferior to those elicited by RSVpreF, BNT162b2, and QIV administered alone. Secondary immunogenicity objectives included demonstrating that immune responses elicited by concomitantly administered RSVpreF and BNT162b2 were noninferior to those elicited by either vaccine alone. Another secondary immunogenicity objective was demonstration that immune responses elicited by concomitantly administered RSVpreF, BNT162b2, and QIV were noninferior to those elicited by RSVpreF, BNT162b2, and QIV administered alone.

The RSV, SARS-CoV-2, and influenza immunogenicity endpoints were evaluated by RSV-A and RSV-B neutralizing titers, SARS-CoV-2 Omicron BA.4/BA.5 and ancestral strain neutralizing titers, and hemagglutination inhibition (HAI) titers for each strain contained in QIV, respectively, (i.e., geometric mean titers (GMTs)) using previously described assays [17,18]. Because GMTs were measured before and 1 month after vaccination, the geometric mean fold rises (GMFRs) were derived as the ratio of the postvaccination result to the prevaccination result as an additional immunogenicity assessment.

### 2.4. Safety Objectives and Assessments

The safety objectives were to describe the safety and tolerability of concomitantly administered and combined RSVpreF and BNT162b2 vaccines given with or without QIV. Frequencies of local reactions and systemic events occurring within 7 days of vaccination were collected by participants using an electronic diary (e-diary). Severity scales for local reactions and systemic events are shown in Table S1. Local reactions were assessed at the injection site on the right arm. Frequencies of adverse events (AEs) and serious AEs (SAEs) occurring through 1 and 6 months after vaccination, respectively, are reported here.

Adverse events of special interest (AESIs) included confirmed diagnoses of myocarditis or pericarditis occurring within 4 weeks after vaccination, or confirmed diagnoses of influenza or RSV infection, or confirmed COVID-19 diagnoses after vaccination through the end of the study (diagnostic procedures are described in the Supplementary Materials).

### 2.5. Statistical Methods

The overall type I error of 5% (2-sided) for the 2 primary immunogenicity objectives was equally split between the primary immunogenicity objectives related to the combined (RSVpreF + BNT162b2) vaccine given with and without QIV (2-sided 2.5% each). The secondary objectives were tested individually after the primary objective was achieved with the same antigen components (i.e., the concomitantly administered RSVpreF/BNT162b2 objective was tested after the combined (RSVpreF + BNT162b2) vaccine given without QIV objective was achieved, and the concomitantly administered RSVpreF/BNT162b2 given with QIV objective was tested after the combined (RSVpreF + BNT162b2) vaccine given with QIV objective was achieved).

For sample size determination, no interference was assumed (i.e., geometric mean ratio (GMR), 1). With 135 evaluable participants per vaccine group, there was ~90% power to declare noninferiority for all 8 antigens (RSV-A, RSV-B, SARS-CoV-2 Omicron BA.4/BA.5, SARS-CoV-2 ancestral strain, and 4 QIV HAI antigens (H1N1 A/Victoria; H3N2 A/Darwin; B/Austria; B/Phuket)) included in the combined (RSVpreF + BNT162b2) vaccine given with QIV, and ~92% power to declare noninferiority for all 4 antigens (RSV-A, RSV-B, SARS-CoV-2 Omicron BA.4/BA.5, and SARS-CoV-2 ancestral strain antigens) included in the combined (RSVpreF + BNT162b2) vaccine given without QIV with a 2-sided 2.5% type-I error. The power for the secondary objectives is similar to that for the primary objectives because the comparisons and assumptions are similar. Assuming 10% of participants were nonevaluable, approximately 150 participants per group were planned to be enrolled.

Immunogenicity assessments were performed in the evaluable immunogenicity population, which included all participants who received the study vaccine to which they were randomized, had blood collection 1 month (27–42 days) after vaccination, had no major protocol deviations, and had at least 1 valid assay result within 1 month after vaccination.

The GMRs, which were defined as the ratio of all the 8 strain/subgroup-specific serology results (measured by RSV neutralizing, SARS-CoV-2 neutralizing, and HAI GMTs) in the concomitantly administered and combined vaccine groups to that for single vaccine administered alone for each of the comparisons, were determined, along with associated 2-sided 97.5% CIs. Using a prespecified 2-fold margin, noninferiority was declared if the lower bound of the 2-sided 97.5% CI for each GMR was greater than 0.5 for RSV-A and RSV-B, SARS-CoV-2 Omicron BA.4/BA.5 and ancestral strains, and all 4 QIV HAI antigens.

The GMTs and corresponding 2-sided CIs were calculated by exponentiating the mean logarithm of the titers and the corresponding CIs determined using the Student *t* distribution. GMFRs were calculated as the group mean of the difference of logarithmically transformed assay results (later timepoint minus earlier timepoint) and exponentiating the mean, and the associated 2-sided 95% CIs were obtained by constructing CIs using the Student *t* distribution for the mean difference on the logarithm scale and exponentiating the confidence limits.

Descriptive summary statistics were determined for local reactions, systemic events, AEs, and SAEs after each vaccination for each vaccine group. Local reactions and systemic events were assessed in the e-diary safety population, which included all participants who received study vaccine and who had at least 1 day of e-diary data transmitted. AEs were assessed in the safety population (i.e., all participants who received study vaccine) and were coded according to the *Medical Dictionary for Regulatory Activities* (v26.0).

## 3. Results

### 3.1. Participants

**Figure 2:**
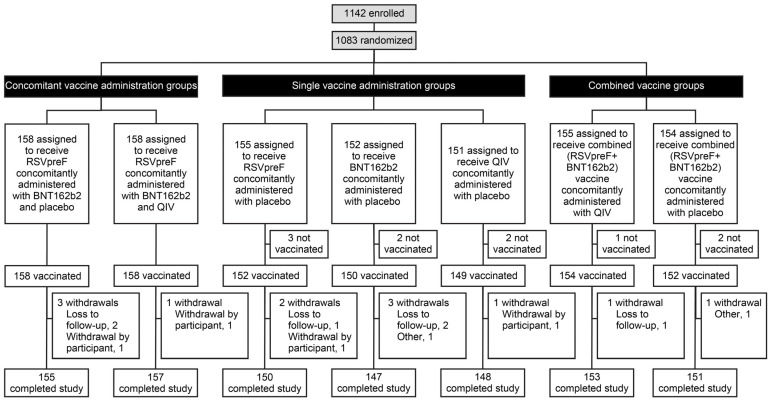
Randomization and vaccine administration. QIV, quadrivalent influenza vaccine.

This study was conducted from 5 June 2023 to 1 January 2024, at 30 sites in the United States. In total, 1083 participants were randomized, and 1073 participants received either concomitantly administered RSVpreF/BNT162b2 (n = 158), concomitantly administered RSVpreF/BNT162b2 given with QIV (n = 158), RSVpreF alone (n = 152), BNT162b2 alone (n = 150), QIV alone (n = 149), combined (RSVpreF + BNT162b2) vaccine given with QIV (n = 154), or combined (RSVpreF + BNT162b2) vaccine alone (n = 152; Figure 2). Overall, 98.1% (1062/1083) of randomized participants completed the 6-month follow-up visit, and 98.0% (1061/1083) completed the study.

Demographic and baseline characteristics of study participants were similar across the seven vaccine groups (Table 1). Overall, 88.9% of participants were White and 89.6% of non-Hispanic/non-Latino ethnicity; median (range) age was 71 (65–90) years. The majority (61.3%) of participants were SARS-CoV-2-positive at baseline. The median time from receipt of the most recent COVID-19 vaccine to study vaccination was 246 days. Overall, 32.5% of participants did not receive an influenza vaccine before study vaccination. Among those who reported previous influenza vaccination, the median number of days from most recent influenza vaccine to study vaccination was 256 days.

### 3.2. Immunogenicity

The evaluable immunogenicity population included 95.9% (1039/1083) of randomized participants. Overall, RSV and SARS-CoV-2 neutralizing GMTs increased from before to 1 month after vaccination for all vaccine groups, and GMFRs trended slightly lower for groups with higher prevaccination GMTs (Figure S1). For the four influenza antigens, HAI GMTs and GMFRs were similar for the three QIV vaccine groups (Figure S2).

**Figure 3:**
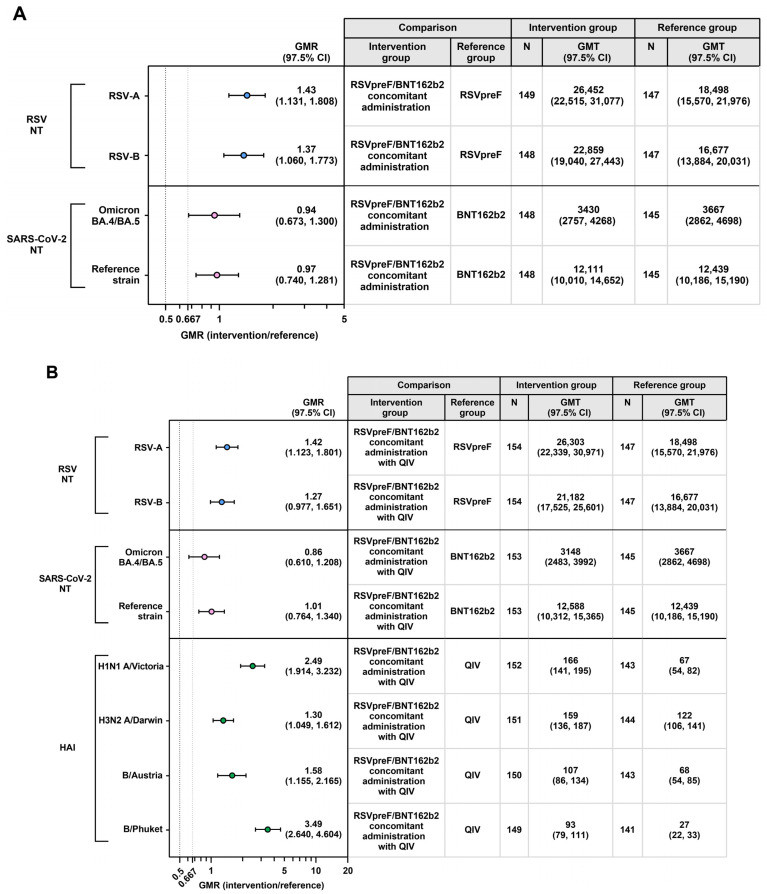
GMRs and GMTs 1 month after vaccination for the comparison of (**A**) RSVpreF concomitantly administered with BNT162b2 versus each vaccine administered alone and (**B**) RSVpreF concomitantly administered with both BNT162b2 and QIV versus each vaccine administered alone. Data are for the evaluable immunogenicity population. The LLOQ values were 10 for HAI titer, 242 for RSV-A NT50, 99 for RSV-B NT50, 71 for SARS-CoV-2 BA.4/BA.5 NT50, and 87 for SARS-CoV-2 ancestral strain. Assay results below the LLOQ were set to 0.5 × LLOQ. GMRs and 2-sided CIs were calculated by exponentiating the mean differences of the logarithms of the titers (assessed group minus comparator group) and the corresponding CIs based on the Student *t* distribution. Using a 2-fold noninferiority margin, success for the immunogenicity objectives was declared if the lower bound of the 2-sided 97.5% CI for the GMR was >0.5. The 2-fold noninferiority margin is denoted with the vertical black dashed line; the additional 1.5-fold noninferiority margin is denoted with the vertical gray dashed line. GMR, geometric mean ratio; GMT, geometric mean titer; HAI, hemagglutination inhibition; LLOQ, lower limit of quantitation; N, number of participants with valid and determinate assay results for the specified assay in the evaluable immunogenicity population; NT50, 50% neutralizing titer; RSV, respiratory syncytial virus; QIV, quadrivalent influenza vaccine.

In the primary immunogenicity analysis comparing RSVpreF concomitantly administered with BNT162b2 to either vaccine administered alone, GMRs 1 month after vaccination were 1.43 (97.5% CI 1.131–1.808) and 1.37 (97.5% CI 1.060–1.773) for the RSV-A and RSV-B neutralizing titers, respectively, and 0.94 (97.5% CI 0.673–1.300) and 0.97 (97.5% CI 0.740–1.281) for the Omicron BA.4/BA.5 and ancestral neutralizing titers, respectively, therefore, meeting the prespecified 2.0-fold noninferiority margin and also meeting a 1.5-fold noninferiority margin (Figure 3A). Similarly, in the comparison of RSVpreF concomitantly administered with both BNT162b2 and QIV, GMRs 1 month after vaccination were 1.42 (97.5% CI 1.123–1.801) for RSV-A and 1.27 (97.5% CI 0.977–1.651) for RSV-B neutralizing titers, 0.86 (97.5% CI 0.610–1.208) for SARS-CoV-2 Omicron BA.4/BA.5 and 1.01 (97.5% CI 0.764–1.340) for ancestral neutralizing titers, and 1.30 (97.5% CI 1.049–1.612) to 3.49 (97.5% CI 2.640–4.604) across strain-specific HAI titers, thus meeting the 2-fold noninferiority margin (Figure 3B). All antigens apart from the SARS-CoV-2 Omicron BA.4/BA.5 strain also met the 1.5-fold noninferiority margin.

**Figure 4:**
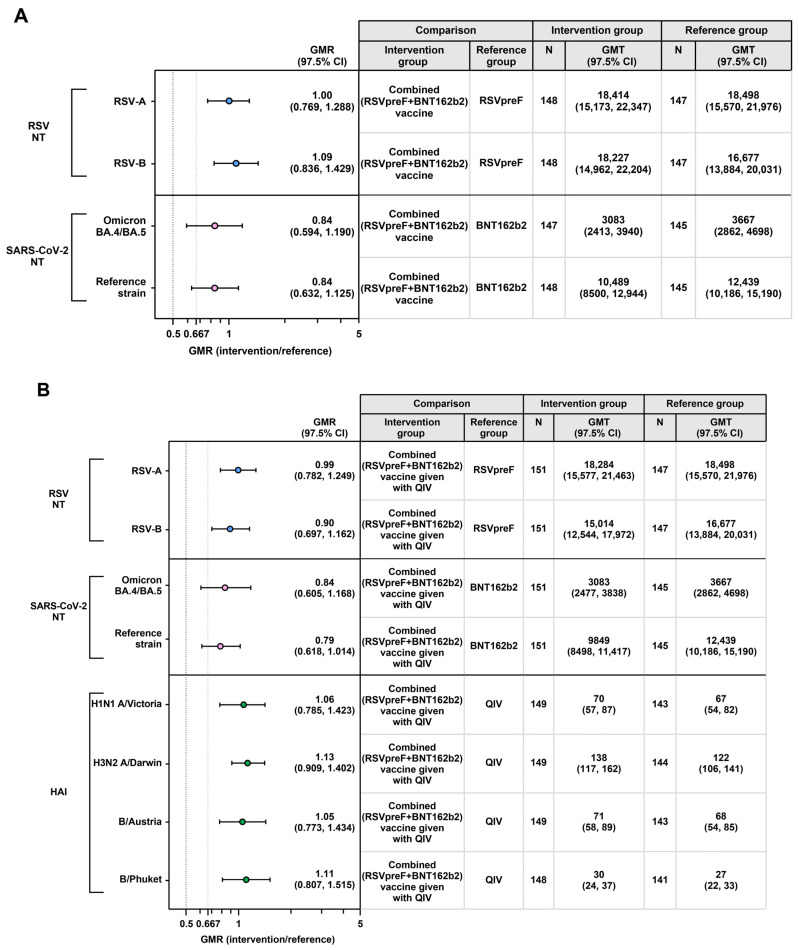
GMRs and GMTs 1 month after vaccination for the comparison of (**A**) combined (RSVpreF + BNT162b2) vaccine concomitantly administered with placebo versus each vaccine administered alone and (**B**) combined (RSVpreF + BNT162b2) vaccine concomitantly administered with QIV versus each vaccine administered alone. Data are for the evaluable immunogenicity population. The LLOQ values were 10 for HAI titer, 242 for RSV-A NT50, 99 for RSV-B NT50, 71 for SARS-CoV-2 BA.4/BA.5 NT50, and 87 for SARS-CoV-2 ancestral strain. Assay results below the LLOQ were set to 0.5 × LLOQ. GMRs and 2-sided CIs were calculated by exponentiating the mean differences of the logarithms of the titers (assessed group minus comparator group) and the corresponding CIs based on the Student *t* distribution. Using a 2-fold noninferiority margin, success for the immunogenicity objectives was declared if the lower bound of the 2-sided 97.5% CI for the GMR was >0.5. The noninferiority margin is denoted with the vertical black dashed line; the additional 1.5-fold noninferiority margin is denoted with the vertical gray dashed line. GMR, geometric mean ratio; GMT, geometric mean titer; HAI, hemagglutination inhibition; LLOQ, lower limit of quantitation; N, number of participants with valid and determinate assay results for the specified assay in the evaluable immunogenicity population; NT50, 50% neutralizing titer; RSV, respiratory syncytial virus; QIV, quadrivalent influenza vaccine.

For both RSV and both SARS-CoV-2 antigens, noninferiority based on the prespecified 2-fold margin (i.e., lower bound of the 2-sided 97.5% CI for each GMR > 0.5) was demonstrated for the combined (RSVpreF + BNT162b2) vaccine to RSVpreF and BNT162b2 administered alone (Figure 4A). Additionally, GMRs 1 month after vaccination for the combined (RSVpreF + BNT162b2) vaccine given with QIV to each of the three vaccines administered alone also met noninferiority based on the prespecified 2-fold margin (Figure 4B). For the combined (RSVpreF + BNT162b2) vaccine given with or given without QIV, GMRs also met the 1.5-fold margin for both RSV antigens and the four QIV strains.

### 3.3. Safety

**Figure 5:**
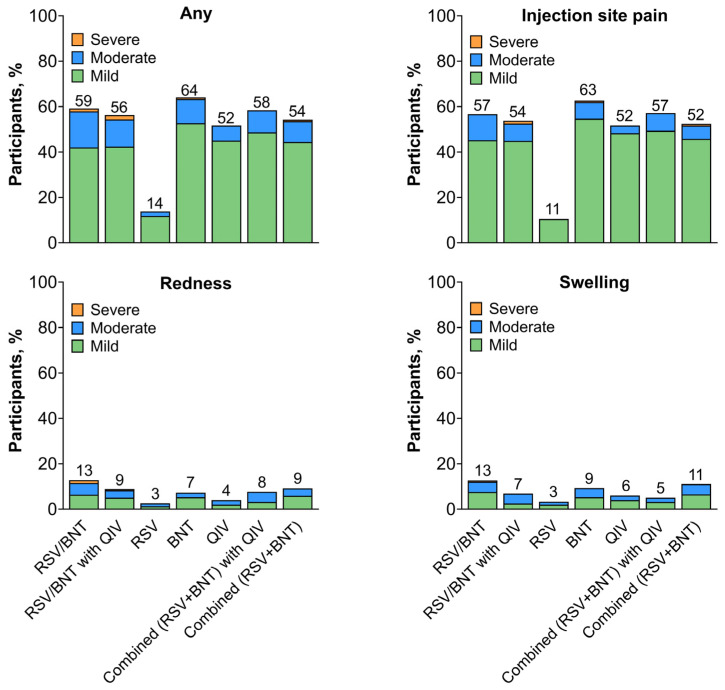
Local reactions. Results are for the electronic diary (e-diary) safety population (i.e., all participants who received the study intervention with ≥1 day of e-diary data transmitted). Severity grading of the specific local reaction is provided in Table S1. Local reactions were assessed at the injection site on the right arm. Numbers above the bars indicate the percentage of participants in each group reporting the specified event (rounded to whole numbers). RSVpreF/BNT, RSVpreF concomitantly administered with BNT162b2 (n = 157) in the right arm and placebo administered in the left arm; RSVpreF/BNT with QIV, RSVpreF concomitantly administered with BNT162b2 in the right arm with QIV administered in the left arm (n = 158); RSVpreF, RSVpreF administered in the right arm and placebo in the left arm (n = 152); BNT, BNT162b2 administered in the right arm and placebo in the left arm (n = 150); QIV, QIV administered in the right arm and placebo in the left arm (n = 149); Combined (RSVpreF + BNT) with QIV, combined (RSVpreF + BNT162b2) vaccine administered in the right arm and QIV administered in the left arm (n = 154); Combined (RSVpreF + BNT), combined (RSVpreF + BNT162b2) vaccine administered in the right arm and placebo in the left arm (n = 153); QIV, quadrivalent influenza vaccine.

For concomitantly administered RSVpreF and BNT162b2 given with or without QIV and the combined (RSVpreF + BNT162b2) vaccine given with or without QIV, frequencies of local reactions (56.3–59.2% and 54.2–58.4%, respectively) were similar to BNT162b2 (64.0%) and QIV (51.7%) administered alone and higher than RSVpreF administered alone (13.8%; Figure 5). Across all vaccine groups, the most common local reaction was injection site pain (51.7–62.7% across all groups apart from the RSVpreF group (10.5%)). Local reactions were mostly mild or moderate in severity. The median onset and duration of local reactions across vaccine groups was 1 to 6 days and 1 to 2.5 days, respectively.

**Figure 6:**
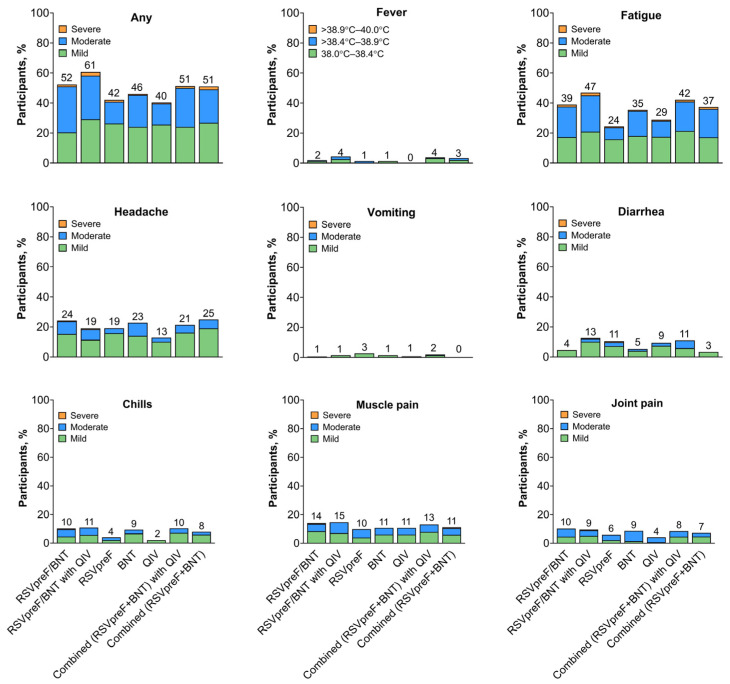
Systemic events. Results are for the electronic diary (e-diary) safety population (i.e., all participants who received the study intervention with ≥1 day of e-diary data transmitted). Severity grading of the specific systemic event is provided in Table S1. Numbers above the bars indicate the percentage of participants in each group reporting the specified event (rounded to whole numbers). RSVpreF/BNT, RSVpreF concomitantly administered with BNT162b2 (n = 157) in the right arm and placebo administered in the left arm; RSVpreF/BNT with QIV, RSVpreF concomitantly administered with BNT162b2 in the right arm with QIV administered in the left arm (n = 158); RSVpreF, RSVpreF administered in the right arm and placebo in the left arm (n = 152); BNT, BNT162b2 administered in the right arm and placebo in the left arm (n = 150); QIV, QIV administered in the right arm and placebo in the left arm (n = 149); Combined (RSVpreF + BNT) with QIV, combined (RSVpreF + BNT162b2) vaccine administered in the right arm and QIV administered in the left arm (n = 154); Combined (RSVpreF + BNT), combined (RSVpreF + BNT162b2) vaccine administered in the right arm and placebo in the left arm (n = 153); QIV, quadrivalent influenza vaccine.

For the concomitant vaccine administration groups and combined vaccine groups, frequencies of systemic events were slightly higher than those across individual vaccine groups (concomitant vaccine groups, 52.2–60.8%; combined vaccine groups, 51.0–51.3%; individual vaccine groups, 40.3–46.0%; Figure 6). Across all vaccine groups, fatigue was the most common systemic event (24.3–46.8% across groups). Systemic events were mostly mild or moderate in severity. Across vaccine groups, the median onset for most systemic events was Day 2, and the median duration was 1 to 2 days.

Adverse events reported for the concomitantly administered RSVpreF and BNT162b2 groups and the combined (RSVpreF + BNT162b2) vaccine groups occurred at similar frequencies to those of the individual vaccine groups (Table 2). Percentages of participants reporting AEs through 1 month after vaccination were 7.2% to 9.1% across vaccine groups. AEs assessed as vaccine related by the investigator were reported in up to 2.5% of participants across all vaccine groups. Three severe AEs were reported (hypertension, ischemic stroke, and injection site hemorrhage in one participant each in the combined (RSVpreF + BNT162b2) vaccine, BNT162b2, and concomitantly administered RSVpreF and BNT162b2 with QIV groups, respectively); the severe AE of right-arm injection site hemorrhage in the participant in the concomitantly administered RSVpreF and BNT162b2 with QIV group was considered vaccine-related by the investigator. No vaccine-related SAEs, life-threatening AEs, deaths, or AEs leading to study withdrawal were reported. No cases of RSV-associated illness, influenza, or the AESIs of confirmed myocarditis or confirmed pericarditis were reported throughout the study (Table S2).

## 4. Discussion

In this study evaluating the safety, tolerability, and immunogenicity of separate and combined RSV and COVID-19 vaccines given with and without QIV, all immunogenicity objectives were met, and all assessed vaccines were safe and well tolerated. The study demonstrated that immune responses elicited by RSVpreF and BNT162b2 administered concomitantly or combined were noninferior to those when the vaccines were administered separately. Immune responses were also noninferior when the three vaccines (RSVpreF, BNT162b2, and QIV) were concomitantly administered. These results support those of previous studies showing the safety and noninferior immunogenicity of concomitantly administered RSVpreF and SIIV [15] and concomitantly administered BNT162b2 and SIIV [16]. Additionally, 1 month after vaccination, immune responses elicited by the combined (RSVpreF + BNT162b2) vaccine given with or without QIV were noninferior to those elicited by the individual vaccines. We also found no new or unexpected safety issues; the safety and tolerability profiles of concomitantly administered RSVpreF and BNT162b2 and the combined (RSVpreF + BNT162b2) vaccine were generally consistent with that of either vaccine given alone and when given with QIV, and it is not anticipated that these investigated regimens will result in appreciable effects on tolerability in their clinical use.

Collectively, these results support receiving RSVpreF and BNT162b2 vaccines together, as well as their administration with QIV, in individuals 65 years and older. Providing RSV and COVID-19 concomitantly administered and combination vaccine regimens alongside QIV in individuals who are recommended to receive all three vaccines may offer convenience to patients and healthcare providers and potentially increase vaccination rates and give maximal protection to these vulnerable populations, and is consistent with current suggestions by the US Centers for Disease Control and Prevention to optimize protection by vaccinating against all recommended seasonal respiratory viruses for indicated individuals [19]. In the United States, adult vaccination rates for COVID-19, influenza, and RSVpreF are suboptimal; as of March 2024, uptake of COVID-19, influenza, and RSV vaccines in adults ≥ 65 years old was 29%, 51%, and 20%, respectively [20,21,22]. It is anticipated that concomitant administration of all three vaccines might increase vaccination rates if individuals were encouraged to do so. Although concomitant vaccine administration is well established in pediatric national immunization programs and for travelers, it is less common in adults; increasing awareness for individuals and healthcare providers on the safety of concomitantly administered and/or combined vaccines may lead to improvements in compliance and uptake rates of routine adult vaccines and decreased use of resources [23].

The admixed combination (RSVpreF + BNT162b2) vaccine given with or without QIV compared with the three vaccines given alone met the primary noninferiority immunogenicity objective and was safe and tolerable. A consideration in the feasibility of the clinical implementation of a combined vaccine for RSV and COVID-19 is the duration of protection afforded against RSV and SARS-CoV-2, respectively. The rapid evolution of SARS-CoV-2 strains, with increased transmissibility and potentially virulence, and waning of protection after vaccination, has necessitated regular (e.g., yearly) updates to the targeted strain within the vaccine, with monovalent JN.1 and KP.2 vaccines now recommended [24,25]. In contrast, RSV does not undergo substantial antigenic drift, with RSV-A and RSV-B as the two cocirculating major antigenic subgroups [9,26]. Data from the pivotal phase 3 efficacy study of RSVpreF indicate that protection against RSV in older adults is sustained for at least two RSV seasons (vaccine efficacy of 81.5% against RSV-associated LRTI) [27]. The need for and timing of RSVpreF revaccination is currently being assessed, but in contrast to COVID-19 and influenza vaccinations, does not appear to require regular (i.e., annual) administration.

One limitation of this study is that it evaluated adults 65 years and older; however, we expect similar results would be observed in individuals from 60 years of age who are eligible to receive RSVpreF. Another study limitation is the exclusion of immunocompromised individuals. Additionally, the BA.4/BA.5 bivalent BNT162b2 vaccine was used in this study, consistent with available recommendations during the study conduct, but is now no longer used in most countries because of more recent variant-adapted versions of the BNT162b2 vaccine being available. Finally, a 2-fold noninferiority margin was used, compared with a 1.5-fold margin typical for concomitant studies. Had the study been powered with a 1.5-fold margin, a larger sample size would have been planned, which would have resulted in greater precision to reject the null hypothesis. Given all influenza strains and RSV subgroups met the 1.5-fold margin, with the lowest GMR point estimate of 0.90 (97.5% CI 0.697–1.162), we expect that the 1.5-fold margin would have been met with a larger sample size.

## 5. Conclusions

This study met all immunogenicity endpoints, demonstrating that immune responses of concomitantly administered RSVpreF and BNT162b2 and combined RSVpreF and BNT162b2 vaccines, given with or without QIV, were noninferior to the administration of separate vaccines. Concomitantly administered vaccines and the combined vaccine, alone or with QIV, were also safe and well tolerated. Collectively, these results support administration of these vaccine regimens in older adults to help protect against these important respiratory pathogens in this vulnerable population.

## Figures and Tables

**Table 1:** Demographic and baseline characteristics.

	Concomitant Vaccine Administration Groups		Single Vaccine Administration Groups			Combined Vaccine Administration Groups	
	RSVpreF/ BNT162b2 (N = 157)	RSVpreF/ BNT162b2 with QIV (N = 158)	RSVpreF (N = 152)	BNT162b2 (N = 150)	QIV (N = 149)	Combined (RSVpreF + BNT162b2) Vaccine with QIV (N = 154)	Combined (RSVpreF + BNT162b2) Vaccine (N = 153)
Sex, n (%)							
Male	63 (40.1)	75 (47.5)	72 (47.4)	70 (46.7)	70 (47.0)	67 (43.5)	60 (39.2)
Female	94 (59.9)	83 (52.5)	80 (52.6)	80 (53.3)	79 (53.0)	87 (56.5)	93 (60.8)
Race, n (%)							
White	139 (88.5)	138 (87.3)	140 (92.1)	131 (87.3)	135 (90.6)	134 (87.0)	137 (89.5)
Black	14 (8.9)	13 (8.2)	10 (6.6)	13 (8.7)	9 (6.0)	10 (6.5)	12 (7.8)
Asian	3 (1.9)	5 (3.2)	1 (0.7)	2 (1.3)	3 (2.0)	8 (5.2)	4 (2.6)
Other * or not reported	1 (0.6)	2 (1.2)	1 (0.7)	4 (2.7)	2 (1.3)	2 (1.3)	0
Ethnicity, n (%)							
Hispanic/Latino	18 (11.5)	15 (9.5)	17 (11.2)	10 (6.7)	12 (8.1)	21 (13.6)	15 (9.8)
Non-Hispanic/non-Latino	138 (87.9)	142 (89.9)	135 (88.8)	139 (92.7)	137 (91.9)	133 (86.4)	137 (89.5)
Not reported	1 (0.6)	1 (0.6)	0	1 (0.7)	0	0	1 (0.7)
Age at vaccination, years							
Mean (SD)	71.4 (4.87)	71.6 (4.79)	72.1 (4.71)	71.0 (4.62)	72.3 (5.12)	71.8 (5.36)	71.7 (5.02)
Median (range)	70 (65–87)	71 (65–90)	71 (65–85)	70 (65–87)	71 (65–87)	70 (65–89)	71 (65–88)
Prior SARS-CoV-2 exposure †, n (%)							
Positive from baseline	97 (61.8)	94 (59.5)	95 (62.5)	93 (62.0)	98 (65.8)	92 (59.7)	89 (58.2)
Positive from 1 month after vaccination	98 (62.4)	96 (60.8)	89 (58.6)	91 (60.7)	96 (64.4)	92 (59.7)	86 (56.2)
Positive from baseline and 1 month after vaccination	96 (61.1)	91 (57.6)	87 (57.2)	88 (58.7)	96 (64.4)	91 (59.1)	84 (54.9)
Days from most recent COVID-19 vaccine to study vaccination							
Mean (SD)	248.7 (51.5)	246.1 (38.0)	240.4 (33.2)	236.4 (33.6)	236.3 (31.7)	238.0 (33.8)	235.8 (38.3)
Median (range)	252 (150–596)	254 (152–390)	246.5 (154–288)	246.5 (150–286)	240 (150–339)	243.5 (141–285)	238 (152–467)
No previous influenza vaccination, n (%)	65 (41.4)	68 (43.0)	37 (24.3)	38 (25.3)	49 (32.9)	44 (28.6)	48 (31.4)
Days from most recent influenza vaccine to study vaccination							
Mean (SD)	284.0 (104.9)	285.0 (75.5)	256.5 (42.7)	253.4 (43.9)	253.4 (39.8)	252.8 (30.2)	257.6 (69.7)
Median (range)	265.5 (181–998)	268.5 (179–632)	256 (133–614)	253 (175–635)	252 (168–560)	256 (138–347)	254 (122–650)

QIV, quadrivalent influenza vaccine; SD, standard deviation. Data are for the safety population (i.e., all participants who received the study intervention). * Includes multiracial, American Indian, Alaska Native, Native Hawaiian, or other Pacific Islander. † Based on N-binding antibody result from baseline or at the 1-month postvaccination visit.

**Table 2:** Adverse events reported through 1 month and serious adverse events reported through 6 months after vaccination.

Adverse Event Type	Concomitant Vaccine Administration Groups		Single Vaccine Administration Groups			Combined Vaccine Administration Groups	
	RSVpreF/ BNT162b2 (N = 157)	RSVpreF/ BNT162b2 with QIV (N = 158)	RSVpreF (N = 152)	BNT162b2 (N = 150)	QIV (N = 149)	Combined (RSVpreF + BNT162b2) Vaccine with QIV (N = 154)	Combined (RSVpreF + BNT162b2) Vaccine (N = 153)
Any AE, * n (%)	14 (8.9)	14 (8.9)	11 (7.2)	12 (8.0)	12 (8.1)	14 (9.1)	13 (8.5)
Related ^†^	4 (2.5)	4 (2.5)	1 (0.7)	1 (0.7)	2 (1.3)	2 (1.3)	2 (1.3)
Immediate ^‡^	0	1 (0.6)	0	0	0	0	1 (0.7)
Severe	0	1 (0.6)	0	1 (0.7)	0	0	1 (0.7)
Life-threatening	0	0	0	0	0	0	0
AESI ^§^	4 (2.5)	1 (0.6)	1 (0.7)	3 (2.0)	0	0	0
Any SAE, ^|^ n (%)	1 (0.6)	3 (1.9)	2 (1.3)	4 (2.7)	2 (1.3)	1 (0.6)	2 (1.3)
Related SAE ^†^	0	0	0	0	0	0	0
Any AE leading to withdrawal, n (%)	0	0	0	0	0	0	0
AESI, ^§|^ n (%)	5 (3.2)	4 (2.5)	2 (1.3)	9 (6.0)	3 (2.0)	2 (1.3)	6 (3.9)
Death, ^|^ n (%)	0	0	0	0	0	0	0

AE, adverse event; AESI, adverse event of special interest; QIV, quadrivalent influenza vaccine; SAE, serious adverse event. Data are for the safety population (i.e., all participants who received the study intervention). * Through 1 month after vaccination. ^†^ As assessed by the investigator. ^‡^ Within 30 min of vaccination. ^§^ AESIs were predefined in the protocol (confirmed diagnosis of influenza, RSV infection, COVID-19, or myocarditis or pericarditis within 4 weeks of vaccination).^|^ Through 6 months after vaccination.

## Data Availability

Upon request, and subject to review, Pfizer will provide the data that support the findings of this study. Subject to certain criteria, conditions, and exceptions, Pfizer may also provide access to the related individual de-identified participant data. See https://www.pfizer.com/science/clinical-trials/trial-data-and-results (accessed on 28 January 2025) for more information.

## Supplementary Materials

The following supporting information can be downloaded at: https://www.mdpi.com/article/10.3390/vaccines13020158/s1, Supplementary Text: Eligibility criteria; Monitoring of potential myocarditis or pericarditis; Diagnosis of COVID-19, RSV-associated illness, and influenza. Figure S1: GMTs for RSV and SARS-CoV-2 50% neutralizing titers before and 1 month after vaccination. Figure S2: HAI GMTs before and 1 month after vaccination. Table S1: Severity scales for local reactions and systemic events. Table S2: Adverse events of special interest reported throughout the study.
