# Supplementary material for: Safety and Immunogenicity of Concomitant Administration and Combined Administration of Bivalent BNT162b2 COVID-19 Vaccine and Bivalent RSVpreF Respiratory Syncytial Virus Vaccine with or Without Quadrivalent Influenza Vaccine in Adults ≥ 65 Years of Age
Source: Vaccines (Basel). 2025 Feb 5;13(2):158. doi: 10.3390/vaccines13020158 (PMC11860858; doi:10.3390/vaccines13020158)
Supplement: Supplementary file 1 [file vaccines-13-00158-s001.zip › Figure S2.pdf]

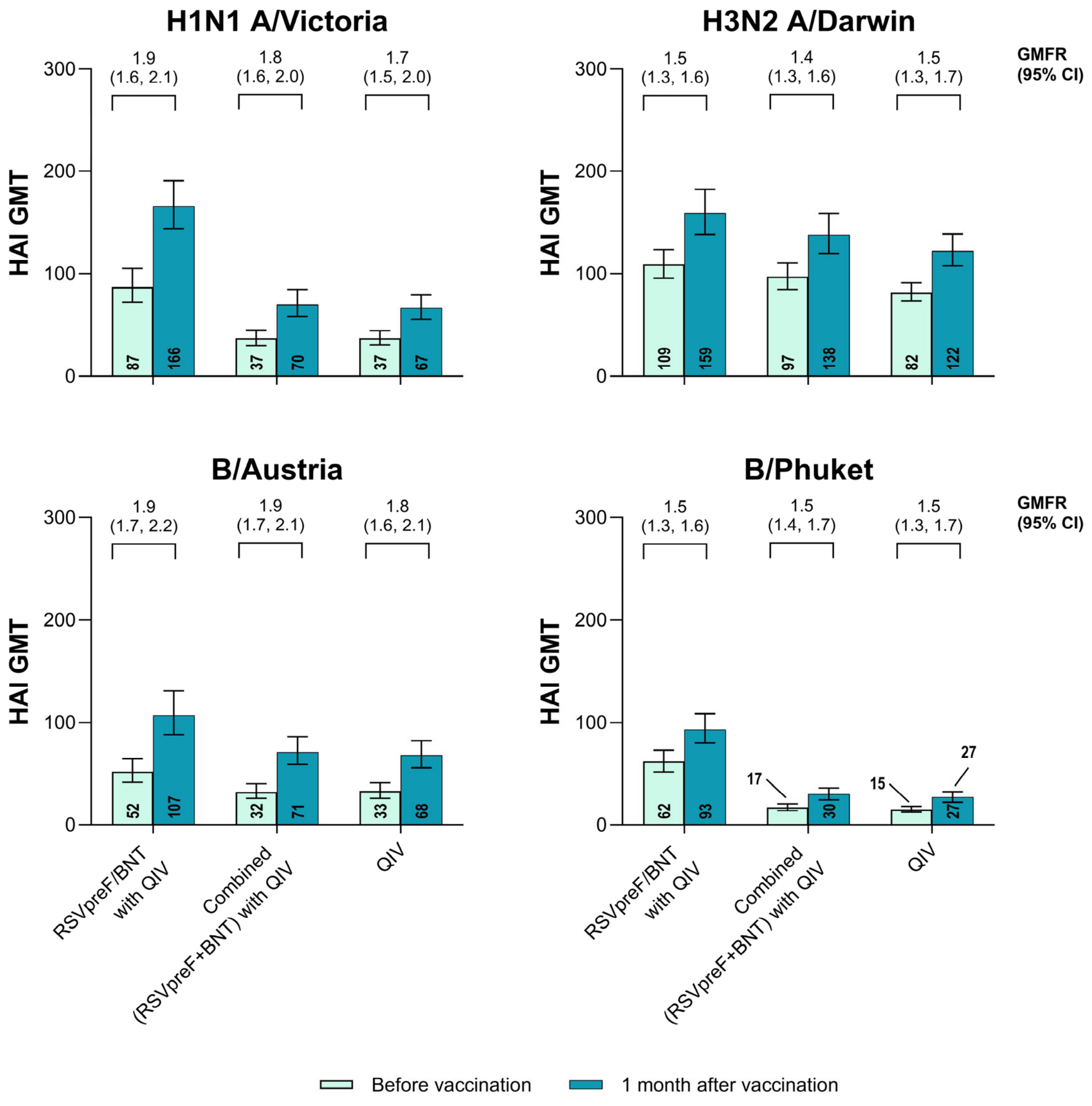

Figure S2. HAI GMTs before and 1 month after vaccination

Data are for the evaluable immunogenicity population. The LLOQ value was 10 for HAI titer. Assay results <LLOQ were set to 0.5 × LLOQ for all GMTs and GMFRs calculation, except when the prevaccination assay result was <LLOQ while the postvaccination result was ≥LLOQ, in which case the prevaccination value was set to LLOQ when calculating GMFRs. RSVpreF/BNT with QIV, RSVpreF concomitantly administered with BNT162b2 in the right arm with QIV administered in the left arm; QIV, QIV administered in the right arm and placebo in the left arm; Combined (RSVpreF+BNT) with QIV, combined (RSVpreF+BNT162b2) vaccine administered in the right arm and QIV administered in the left arm. GMFR, geometric mean fold rise (from before to 1 month after vaccination); GMT, geometric mean titer; HAI, hemagglutination inhibition; LLOQ, lower limit of quantitation; QIV, quadrivalent influenza vaccine.
