# Supplementary material for: Safety and Immunogenicity of Concomitant Administration and Combined Administration of Bivalent BNT162b2 COVID-19 Vaccine and Bivalent RSVpreF Respiratory Syncytial Virus Vaccine with or Without Quadrivalent Influenza Vaccine in Adults ≥ 65 Years of Age
Source: Vaccines (Basel). 2025 Feb 5;13(2):158. doi: 10.3390/vaccines13020158 (PMC11860858; doi:10.3390/vaccines13020158)
Supplement: Supplementary file 1 [file vaccines-13-00158-s001.zip › Supplementary Text.pdf]

SUPPLEMENTARY TEXT

Eligibility Criteria

Inclusion criteria included healthy participants with preexisting stable disease, defined as disease not requiring significant change in therapy or hospitalization for worsening disease during the 6 weeks before enrollment, could be included.

Key exclusion criteria included:

- Confirmed diagnosis of COVID-19, RSV infection, or influenza ≤120 days before study intervention administration
- History of severe adverse reaction associated with any vaccine and/or severe allergic reaction (eg, anaphylaxis) to any component of the study intervention(s)
- Immunocompromised individuals with known or suspected immunodeficiency
- Bleeding diathesis or condition associated with prolonged bleeding
- Allergy to egg proteins (egg or egg products) or chicken proteins
- Receipt of chronic systemic treatment with known immunosuppressant medications, or radiotherapy, within 60 days before enrollment through conclusion of the study
- Receipt of blood/plasma products, immunoglobulin, or monoclonal antibodies, from 60 days before study intervention administration, or planned receipt throughout the study
- Receipt of any RSV vaccine at any time before enrollment, or planned receipt throughout the study

Monitoring of Potential Myocarditis or Pericarditis

Any study participant who reported acute chest pain, shortness of breath, palpitations, or any other symptom(s) possibly indicative of myocarditis or pericarditis within 28 days after study vaccination was evaluated for possible myocarditis or pericarditis. In addition to a clinical evaluation, an electrocardiogram measurement of troponin level was conducted. If myocarditis or pericarditis was suspected based upon the initial evaluation, the following was also performed:

- Evaluation by a cardiologist,
- Cardiac echocardiogram, and/or
- Cardiac magnetic resonance study

Diagnosis of COVID-19, RSV-associated illness, and influenza

COVID-19 was diagnosed by clinical signs or symptoms[1] and positive SARS-CoV-2 nucleic acid amplification test or rapid antigen test result. RSV-associated illness and influenza were diagnosed clinically by the investigator.

---

[1] Centers for Disease Control and Prevention 2022. Symptoms of COVID-19. Available at: https://www.cdc.gov/coronavirus/2019-ncov/symptoms-testing/symptoms.htm (accessed November 11, 2023)
