# Supplementary material for: Safety and Immunogenicity of Concomitant Administration and Combined Administration of Bivalent BNT162b2 COVID-19 Vaccine and Bivalent RSVpreF Respiratory Syncytial Virus Vaccine with or Without Quadrivalent Influenza Vaccine in Adults ≥ 65 Years of Age
Source: Vaccines (Basel). 2025 Feb 5;13(2):158. doi: 10.3390/vaccines13020158 (PMC11860858; doi:10.3390/vaccines13020158)
Supplement: Supplementary file 1 [file vaccines-13-00158-s001.zip › Table S1.pdf]

Table S1. Severity scales for local reactions and systemic events

	Mild	Moderate	Severe	Grade 4
**Local reactions**				
Injection site pain	Does not interfere with activity	Interferes with activity	Prevents daily activity	ED visit or hospitalization
Redness	>2.0–5.0 cm	>5.0–10.0 cm	>10 cm	Necrosis or exfoliative dermatitis
Swelling	>2.0–5.0 cm	>5.0–10.0 cm	>10 cm	Necrosis
**Systemic events**				
Vomiting	1–2 times in 24 hours	>2 times in 24 hours	Requires IV hydration	ED visit or hospitalization
Diarrhea	2–3 loose stools in 24 hours	4–5 loose stools in 24 hours	≥6 loose stools in 24 hours	ED visit or hospitalization
Headache	Does not interfere with activity	Some interference with activity	Prevents daily routine activity	ED visit or hospitalization
Fatigue/tiredness	Does not interfere with activity	Some interference with activity	Prevents daily routine activity	ED visit or hospitalization
Chills	Does not interfere with activity	Some interference with activity	Prevents daily routine activity	ED visit or hospitalization
Muscle pain	Does not interfere with activity	Some interference with activity	Prevents daily routine activity	ED visit or hospitalization
Joint pain	Does not interfere with activity	Some interference with activity	Prevents daily routine activity	ED visit or hospitalization

ED, emergency department; IV, intravenous.
