# Supplementary material for: Safety and Immunogenicity of Concomitant Administration and Combined Administration of Bivalent BNT162b2 COVID-19 Vaccine and Bivalent RSVpreF Respiratory Syncytial Virus Vaccine with or Without Quadrivalent Influenza Vaccine in Adults ≥ 65 Years of Age
Source: Vaccines (Basel). 2025 Feb 5;13(2):158. doi: 10.3390/vaccines13020158 (PMC11860858; doi:10.3390/vaccines13020158)
Supplement: Supplementary file 1 [file vaccines-13-00158-s001.zip › Table S2.pdf]

Table S2. Adverse events of special interest reported throughout the study

	Concomitant vaccine administration groups		Single vaccine administration groups			Combined vaccine groups	
	**RSVpreF/ BNT162b2 (N=157)**	**RSVpreF/ BNT162b2 with QIV (N=158)**	**RSVpreF (N=152)**	**BNT162b2 (N=150)**	**QIV (N=149)**	**Combined (RSVpreF+BNT162b2) vaccine with QIV (N=154)**	**Combined (RSVpreF+BNT162b2) vaccine (N=153)**
Any AESI, n (%)	5 (3.2)	4 (2.5)	2 (1.3)	9 (6.0)	3 (2.0)	2 (1.3)	6 (3.9)
RSV-associated illness	0	0	0	0	0	0	0
Influenza	0	0	0	0	0	0	0
COVID-19	4 (2.5)	4 (2.5)	1 (0.7)	9 (6.0)	3 (2.0)	2 (1.3)	6 (3.9)
SARS-CoV-2 positive	2 (1.3)	0	1 (0.7)	3 (2.0)	0	0	1 (0.7)
Myocarditis or pericarditis	0	0	0	0	0	0	0

AESI, adverse event of special interest; QIV, quadrivalent influenza vaccine.
